# Assessing Student’s Achievement Gaps between Ethnic Groups in Brazil

**DOI:** 10.3390/jintelligence7010007

**Published:** 2019-02-20

**Authors:** Luis E. C. Rocha, Luana de F. Nascimento

**Affiliations:** 1 Department of International Business and Economics, University of Greenwich, London SE10 9LS, UK; 2 SCK-CEN Belgian Nuclear Research Centre, Boeretang 200, 2400 Mol, Belgium; ldfnasci@sckcen.be

**Keywords:** achievement gap, attainment gap, ethnic students, secondary school assessment

## Abstract

Achievement gaps refer to the difference in the performance on assessments of students belonging to different social groups. Achievement gaps between ethnic groups have been observed in countries with heterogeneous populations. In this paper, achievement gaps between ethnic populations in Brazil were analyzed by studying the performance of a large cohort of senior high-school students in a standardized national exam. Ethnic groups were stratified by Brazilian states and socio-economic variables to homogeneize the groups, and the analyses focused on the disciplines of mathematics and writing that involve different cognitive functions. A Welch’s *t*-test analysis was performed and key socio-economic variables that may explain the gaps were studied. The results show that gaps between ethnic groups of students living in low-income households were either statistically insignificant or small (2–6%) if statistically significant. Larger gaps however were observed for students coming from high-income families in some contexts. Although parental education was associated with higher performance, it may either increase, decrease or maintain the gaps between White and Black, and between White and Pardo students. Our results support that socio-economic variables, linked to historical developments, have an impact on student’s performance irrespective of ethnic background, resulting on little to no influence on group performance when students are exposed to similar cultural and economic contexts.

## 1. Introduction

Education is the systematic process of sharing knowledge and developing skills among people [1,2]. It is generally seen as a fundamental right of citizens [3] but also as an asset both individually and for the country [4]. Nevertheless, funding is typically limited and several countries struggle to improve their formal educational systems to provide quality education [5,6]. In several cases, strategies are sometimes absent or not well-defined and the already scarce resources end up sub-optimally allocated. A major challenge is to provide similar education opportunities to everyone, given the heterogeneity of students [7] and resources available at different locations. Moreover, even if biologically different, people are also affected in daily life by the socio-economic context in which they live and thus students may have their own individual struggles shaping their learning process.

Examinations are commonly used to assess student’s learning. Scores are then used to rank individuals and to compare the performance of groups of students, such as entire schools, cities, countries, income, ethnicities, sex or other demographic groups. The achievement gap is a term generally used to quantify the persistent difference in the performance of students belonging to different groups in standardized tests [8]. Much attention has been given to achievement gaps between male and female, low- and high-income, and white and non-white students [9,10]. In particular, achievement gaps between ethnic groups, as estimated through formal examinations, exist in several countries but the gap size is not consistent across countries and ethnic groups [11,12,13,14]. Such gap analysis should not be used to stigmatize groups but to better understand needs and to develop target interventions and policies, aiming to improve education of group members and consequently homogenize performance of the entire population to higher levels. The achievement gap is in the political and academic agenda. Although gaps between various groups remain prevalent worldwide [9,10,15,16,17], several experiences have shown that it is possible to homogenize student’s performance irrespective of their background [18,19].

Brazil, like several other countries particularly in the Americas, has a relatively recent history of colonization involving spontaneous (in particular, European and Asian) and forced migration (through slavery), and tentative integration with indigenous populations. Although mixing between ethnic groups is generally perceived as higher than in some other countries with similar migration patterns, several indicators suggest a level of segregation by ethnicity, particularly of so-called whites (typically of European ancestry), blacks (typically descendants of enslaved populations from sub-Saharan Africa between the mid-15th and mid-19th century) and mixed-ethnicities (typically those with black and white ancestry, but also white and indigenous, among other combinations), when it comes to income, education, health, and job opportunities [20,21,22,23,24,25]. Income is perceived as a strong indicator of segregation in Brazilian society, where poorer populations are marginalized with less access to essential services, entertainment and culture. This country-wide segregation creates power relations and leads to stigma since poverty, criminality, performance or cognitive capacity are associated with particular groups. Aiming to reduce educational gaps of students with diverse income and ethnic backgrounds, governmental affirmative actions have been implemented at higher education in the last decade [26,27] but relatively few efforts have been done at the primary and secondary education levels [28,29].

In this paper, we analyzed achievement gaps between ethnic populations in Brazil by studying the performance of a large cohort of senior high-school students in a standardized national exam. We hypothesize that although individual performance may differ, gaps between groups are mostly driven by socio-economic variables with close links to historical developments that limited access to quality education for groups of students. Students exposed to similar socio-economic contexts should perform similarly, irrespective of their ethnic background. To perform a fair statistical analysis, one fundamental strength of our study is that we homogenized the ethnic groups by removing potential geographic biases associated with the availability of infrastructure and resources, socio-economic and cultural backgrounds, and some vulnerable populations that may be over-represented in some ethnic groups. We also focused our analysis on mathematics and writing that are relevant core disciplines involving different cognitive functions to assess the universality of the results. We estimated the achievement gaps and their statistical significance through the advanced Welch’s analysis of variance and investigated key socio-economic factors that may explain the existence or absence of gaps in the various cases.

## 2. Materials and Methods

### 2.1. Data

The data corresponds to the performance scores of students in the Brazilian national high school (secondary education) exam (abbreviated “ENEM” from the Portuguese “Exame Nacional do Ensino Médio”) [30,31]. This is a standardized national exam, developed and coordinated by the Federal Ministry of Education of Brazil. Since its first edition in 1998, the national exam occurs every year. Although non-mandatory, the individual scores can be currently used by students to compete for enrollment at federal universities (tuition-free) and for other scholarship programs to study in private universities (with tuition). Any person at any age who has completed or is about to complete secondary education can participate in the exam. The government also uses the results to evaluate the quality of education country-wide. Datasets containing information for each year are publicly available and can be freely downloaded from “http://portal.inep.gov.br/microdados”.

The exam is divided in two stages taking place on two different days approximately by the end of the Brazilian academic year, which is in late October or early November. The first stage lasts a maximum of four and a half hours and contains 45 multiple choice questions of Natural Sciences (i.e., Biology, Physics and Chemistry) and 45 multiple choice questions of Social Sciences (i.e., History, Geography, Philosophy and Sociology). The second stage lasts a maximum of five and a half hours and contains 45 multiple choice questions of Languages (i.e., Portuguese grammar, Literature, Spanish or English, Arts, Physical Education, and Information and Communication Technologies), 45 multiple choice questions of Mathematics, and 1 written essay (hereafter Writing exam) with a surprise topic typically on current issues. Each exam has four versions that are randomly distributed among the students to avoid cheating but the written essay has a common topic. The multiple choice question scoring is based on the Item response theory that takes into account the level of difficulty of each question and the response patterns of students, giving lower weight to potential guessing [32,33]. On the other hand, the written essay is graded (and scores are then averaged) by two independent examiners following five criteria of competences related to text interpretation, organization of ideas, grammar, topic, ethics and so on [34].

### 2.2. Ethnic Structure

Ethnic and racial classifications are difficult to standardize and generally vary across countries. In Brazil, the standard is to follow the classification made by The Brazilian Institute of Geography and Statistics (abbreviated IBGE in Portuguese www.ibge.gov.br), which is a federal agency responsible for the national census and other demographic and socio-economic official surveys of the Brazilian population. The IBGE classifies Brazilians according to self-declared “skin-color” as “Branco” (White, mostly of European ancestry but also Middle Easters), “Negro” (Black, typically of sub-Saharan African ancestry), “Pardo” (that has broad meaning and includes miscegenation of white and black people, white and indigenous, and black and indigenous; information about the ethnicity of parents is unavailable), “Amarelo” (literally meaning Yellow, corresponding to Asians, from East Asia, particularly Japan, Korea and China) and “Indigena” (Indigenous people). In the latest official census before the examination data used in this study, i.e., from 2010, 47.7% of the population self-declared White, 7.6% Black, 43.1% Pardo, 1.1% Asian, 0.4% Indigenous, and 0.7% none [35]. This distribution varies widely across the country. In the national exam ENEM, participants also self-declare as belonging to one of these ethnic groups and this self-classification will be used in our analyses.

### 2.3. Inclusion Criteria

We used data from the exam that occurred on the 8th and 9th of November, 2014. Given the diversity of the demographic and socio-economic background of students, we chose inclusion criteria to homogenize the sample and minimize spurious influences of external factors and confounding variables, yet aiming for large samples. Therefore, in our sample the student must (i) have self-declared ethnicity; (ii) be born and raised in Brazil; (iii) have followed most of the secondary education in publicly administered and funded schools; (iv) have completed secondary education in 2014 and have 17 (seventeen) years old (this is the expected age to complete secondary education); (v) have participated in both parts of the exam; (vi) not be disabled; (vii) not be pregnant or lactating; (viii) not be married, divorced or widowed; (ix) live in an urban area; (x) study in an urban school; (xi) live in a house with 6 (six) or less people including the candidate. Criteria *i* was used to guarantee information about ethnicity; criteria *ii* was used to guarantee that the participant was enrolled in the Brazilian educational system from the early age and criteria *iii* guaranteed that the student’s recent education was in public schools (rather than the more exclusive private institutions); criteria *iv* guaranteed comparison between students at the same level and age; criteria *v* was used to filter participants with performance scores available for all exams; criteria *vi*–*xi* were used to remove vulnerable students from the sample since these students, for different reasons, have disadvantages in terms of available time for studies, physical or mental limitations, and family support in case of large families, and may be overly represented in some ethnic or socio-economic groups. For example, we calculated that 16.5% of the entire student population is married, young mothers or rural. In the low-income bracket, 27.3% of the students are White, 22.1% Black, 28.4% Pardo, 24.1% Asian and 32.7% Indigenous. On the other hand, only 8.0% is White, 5.3% Black, 7.0% Pardo, 6.5% Asian and 15.4% Indigenous are high-income. The possibility to homogenize the population characteristics to remove such differences is a particular strength of our study. Our final sample has 388,564 students. The variable names (codes) for these inclusion criteria as available in the original data set are specified in the Supplementary Materials (Table S1).

### 2.4. Statistical Analysis

To statistically assess if the population mean scores for the different ethnic groups differ, we performed a Welch’s Analysis of Variance (ANOVA) test between the means of all ethnic groups. ANOVA splits the aggregate variability found inside a data set into systematic factors that have statistical influence on the data, and random factors that have no influence, therefore providing ways to statistically infer the significance of differences in the means. The null hypothesis in our study is that any difference between the ethnic groups is due to chance, i.e., $H_{0}:x_{\mathrm{white}}=x_{\mathrm{black}}=x_{\mathrm{pardo}}=x_{\mathrm{asian}}=x_{\mathrm{indigenous}}$. The alternative hypothesis is $H_{1}:x_{\mathrm{white}}\neq x_{\mathrm{black}}\neq x_{\mathrm{pardo}}\neq x_{\mathrm{asian}}\neq x_{\mathrm{indigenous}}$. Since this test only indicates if at least two means are different, we also apply the Welch’s *t*-test for pairs of ethnic groups such that $H_{0}:x_{\mathrm{group}-1}=x_{\mathrm{group}-2}$ and $H_{1}:x_{\mathrm{group}-1}\neq x_{\mathrm{group}-2}$. The F-statistic for the Welch’s *t*-test is given by

(1) $$F=\frac{\frac{1}{k-1}\sum_{j=1}^{k}w_{j}{\left({\bar{x}}_{j}-{\bar{x}}^{\prime }\right)}^{2}}{1+\frac{2(k-2)}{k^{2}-1}\sum_{j=1}^{k}\left(\frac{1}{n_{j}-1}\right){\left(1-\frac{w_{j}}{w}\right)}^{2}},$$

where *k* is the number of ethnic categories, $n_{j}$ is the size of each category and

(2) $${\bar{x}}_{j}=\frac{1}{n_{j}}\sum_{i=1}^{n_{j}}x_{i},\;s_{j}^{2}=\frac{1}{n_{j}-1}\sum_{i=1}^{n_{j}}{\left(x_{i}-{\bar{x}}_{j}\right)}^{2},\;$$

(3) $$w_{j}=\frac{n_{j}}{s_{j}^{2}},\;w=\sum_{j=1}^{k}w_{j},\;{\bar{x}}^{\prime }=\frac{1}{w}\sum_{j=1}^{k}w_{j}{\bar{x}}_{j},$$

that is, ${\bar{x}}_{j}$ and $s_{j}^{2}$ are respectively the mean and variance in each ethnic category *j*. Therefore

(4) F∼F(k-1,df),

where the degrees of freedom are given by

(5) $$df=\frac{k^{2}-1}{3\sum_{j=1}^{k}\left(\frac{1}{n_{j}-1}\right){\left(1-\frac{w_{j}}{w}\right)}^{2}}.$$

The Welch’s statistical test is more robust and recommended if samples have different sizes and variances as the case of our data, but the test also performs well otherwise [36,37,38]. We chose a strong level of confidence α=0.99. Therefore, if the *p*-value < 0.01 we rejected the null hypothesis and concluded that there was a significant difference between the mean scores. On the other hand, *p*-values ≥ 0.01 indicated that the null hypothesis was true, i.e., mean scores were equal. The achievement gap was given by $\Delta =\left({\bar{x}}_{\mathrm{group}-1}-{\bar{x}}_{\mathrm{group}-2}\right)/{\bar{x}}_{\mathrm{group}-2}$, that is, the percentage of the difference between two ethnic groups.

## 3. Results

Brazil is a federative republic with 23 states plus the federal district where the capital of the country is located. Education in Brazil is mostly publicly funded and free, where 78.5% of the primary and 70.8% of the secondary schools are managed by the state [39]. Given that primary and secondary education are mostly financed by the local authorities (respectively at the municipal and state levels) and sociocultural factors and values are also generally defined locally due to the large geographical area of Brazil and ethnic-cultural heterogeneity due to different waves of immigration, we studied each federal state independently. We first analyzed the states of Sao Paulo and Amazonas because their contrasting ethnic, geographic, demographic and socio-economic contexts that generate somewhat opposite ecosystems; and then we studied and compared all Brazilian states independently. The goal of this exercise is to show the universality of our findings since the other federal states generally have characteristics in-between these two study cases. Sao Paulo had a population of 41.262199 people with a White majority in 2010 [35], household income per capita of 1432 BRL (approx. 814 USD) [40] and HDI of 0.819 [41] in 2014. It is highly industrialized and populated, with several cosmopolitan cities with vibrant cultural life, well-connected domestically and internationally, and also has a strong agricultural sector. Amazonas on the other hand had a population of 3.483985 people with a Pardo majority in 2010 [35], household income per capita of 739 BRL (approx. 420 USD) [40] and HDI of 0.709 [41] in 2014. Although about half of the population lived in the capital Manaus, the rest lived in smaller towns with less than approximately 100,000 inhabitants with poor transportation and immersed in the Amazon forest. The economy of the state is mostly based on extractivism with relevant levels of industrialization in the capital. For each state, we grouped students in 5 categories according to their ethnic background and 6 categories (referred to as G1 to G6) according to their household income level (Section 3.1) or their parental education level (Section 3.2).

### 3.1. Household Income

**Figure 1:**
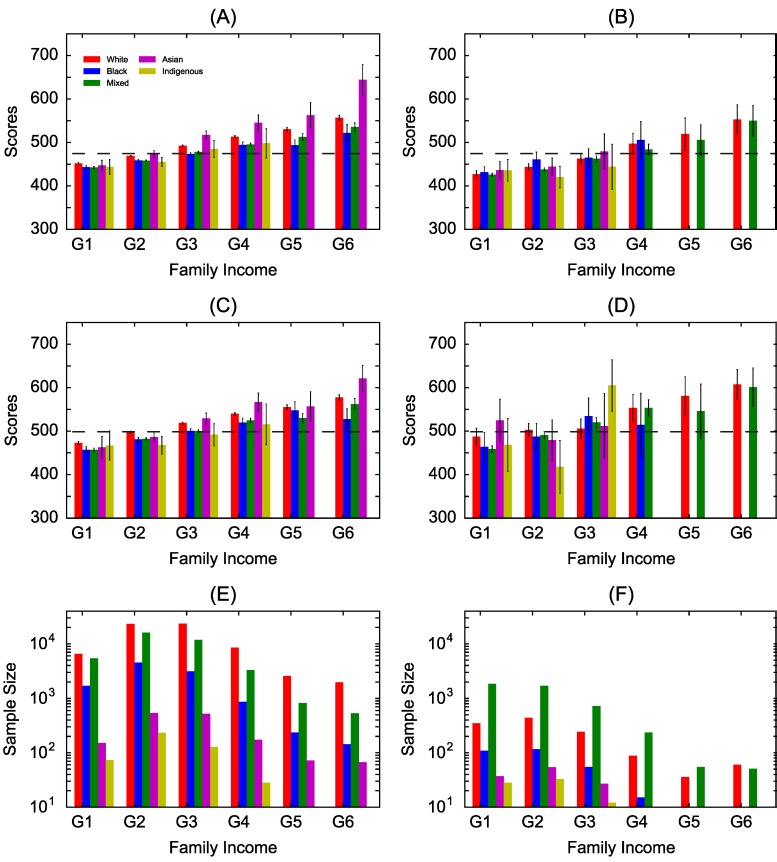
Mean performance score vs. household income level. The mean score for 5 ethnic groups and 6 income levels: (**A**) mathematics in Sao Paulo; (**B**) mathematics in Amazonas; (**C**) writing in Sao Paulo; and (**D**) writing in Amazonas. The sample size for the ethnic groups and income levels: (**E**) Sao Paulo and (**F**) Amazonas. The household income levels correspond to: G1: up to 1 minimum salary (411 USD in 2014); G2: between 1 and 2; G3: between 2 and 4; G4: between 4 and 6; G5: between 6 and 8; and G6: above 8. Black vertical bars represent the 95% confidence intervals and the horizontal dashed lines are the national mean for each discipline.

There is generally a trend of increasing mean scores in both mathematics and writing for increasing household income in both states (Figure 1A–D). The trend is less clear for writing in Amazonas (Figure 1D). This is likely due to the relatively small populations in some categories in this state (Figure 1E,F show the samples size in each category) also generate relatively larger variance (black lines). We do not use categories with sample sizes smaller than 10 students. Results for Sao Paulo have less variations of the mean scores and smaller confidence intervals, likely due to the relatively larger sample sizes. In general, the two groups with lowest family income (G1 and G2) have mean scores below the national mean, i.e., considering all states (dashed lines), whereas the two groups with highest household income have mean scores above the national mean. There is also an apparent increasing gap between some ethnic groups as the income increases. For low household income, we observe mixed results with slightly higher scores for one or another ethnic group depending on the discipline and state. In Sao Paulo there is a clear positive trend of increasing gap for increasing income between Asians and White students in comparison to Blacks and Pardos. In the state of Amazonas, some ethnic groups are not representative in high income categories but we generally observe similar mean scores for White and Pardo students, i.e., absence of gaps, with large confidence intervals.

The analysis of all ethnic groups together indicates that in the state of Sao Paulo, there are statistically significant (*p*-value < 0.01) differences in the mean scores among groups whereas no differences are observed in the state of Amazonas (*p*-value ≥0.01) (Table 1). Results are generally similar for both disciplines. For a more detailed analysis, we now look at pairs of ethnic groups taking the White students as the reference group, i.e., we compare each ethnic group against the Whites. The *p*-values indicate statistically significant gaps (Δ) in the mean scores between White (higher mean score) and Black (lower), and between White (higher) and Pardo (lower) students for almost all income levels in both disciplines in the state of Sao Paulo. For low income, this gap is slightly higher for writing (Δ=3.4%) in comparison to mathematics (Δ∼2%). There is also an increasing gap for higher income levels for mathematics but this increase is not evident for writing. White students tend to perform worse than the Asians in mathematics when statistically significant gaps are observed, with a substantial gap increase for higher income in favor of Asian students. In the case of White and Indigenous students, there are typically no statistically significant gaps in mean scores. In the state of Amazonas, we observe no statistical significant gaps with the exception of writing scores for White and Indigenous students. However, the unusual large gap (Δ∼18.4% to Δ=-17.9%) from favoring White to favoring Indigenous students between two subsequent income levels suggest outliers and would need more data for a careful analysis. Looking at the mean scores, we generally identify worse performance (Δ<0) of White students in comparison to Black, Pardo and Asian students, particularly in the case of mathematics.

Analyzing the mean scores of each of the 24 Brazilian federal states independently, there are no statistically significant gaps between ethnic groups in most of the cases for very low and very high income households (See Tables S2 and S3 for all statistical results). Statistically significant differences are observed for very low income in three states (MG, RJ and SP) with better performance of White students in comparison to Blacks and Pardos in mathematics, and better performance of Whites in comparison to Asians in one state (MG). For writing, in a few states White students also showed better performance in comparison to Black, Pardo and Asian students (with gaps slightly larger than in the case of mathematics) but there are generally no strong national trends favoring one or another ethnicity.

### 3.2. Parental Education

**Figure 2:**
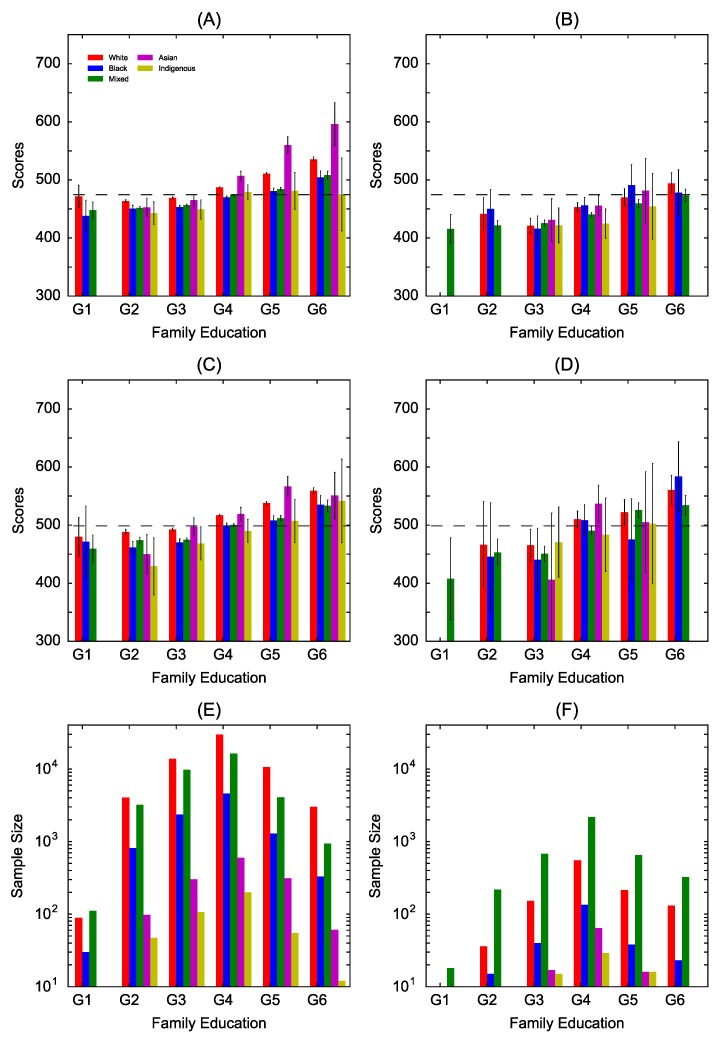
Mean performance score vs. parental education level. The mean score for 5 ethnic groups and 6 parental education levels: (**A**) mathematics in Sao Paulo; (**B**) mathematics in Amazonas; (**C**) writing in Sao Paulo; and (**D**) writing in Amazonas. The sample size for the ethnic groups and parental education levels: (**E**) Sao Paulo and (**F**) Amazonas. The education levels correspond to: G1: both parents have no formal education; G2: at least one parent has completed 4 years of formal education; G3: at least one parent has completed 8 years of formal education (i.e., primary education) or completed primary but not secondary education (may have started but have not completed secondary); G4: at least one parent completed secondary but not tertiary education (may have started but have not completed tertiary); G5: at least one parent completed tertiary education; and G6: at least one parent has completed post-graduate studies. Black vertical bars represent the 95% confidence intervals and the horizontal dashed lines are the national mean for each discipline.

There is a general trend of increasing mean scores for students which parents have more formal education but mean scores do not differ much for groups of students with less educated parents (G1 to G3) (Figure 2A–D). This happens for both disciplines in both states but in Amazonas the confidence intervals are generally larger (Figure 2B,D). In Sao Paulo, White students tend to perform better than other ethnic groups with the exception that Asian students perform substantially better in mathematics in case of highly educated parents (Figure 2A) and also in group G5 in the case of writing (Figure 2C). In the state of Amazonas, there is a mixed pattern in which the best performance alternates between ethnic groups, including for highly educated parents, for both disciplines (Figure 2B,D). Note also that the distribution of the sample sizes are higher in mid-level education (G4) in both states (Figure 2E,F) whereas we observe larger samples sizes in lower household income levels in Figure 1E,F.

The analysis of all mean scores together indicates that the gap is statistically significant for both disciplines in Sao Paulo but not in the state of Amazonas (Table 2). Looking at pairs of ethnic groups, the gap is statistically significant (*p*-value < 0.01) between White (higher mean scores) and Black (lower) students, and White (higher) and Pardo (lower) students for all levels but G1. Furthermore, in highly educated families, there is also statistical significance between White and Asian students, particularly in mathematics, with Asian students achieving higher mean scores. Generally speaking, no statistically significant gaps are observed between White and Asian students. In the case of Amazonas, there is no statistical significant differences, with the exception of White and Black in G4 for mathematics. In the lower end of the spectrum (i.e., low parental education), the gaps (Δ) between ethnic groups are generally higher in the respective educational level in comparison to the gaps observed in the categories defined by low household income, whereas the opposite occurs in the higher end, i.e., the gap is relatively smaller in highly educated families than in families with higher income levels. In other words, household income seems to be a stronger indicator (than parents education) of higher scores. Our statistical analysis further indicates that in the state of Amazonas, there are no clear trends in the gaps, i.e., for a given parental education level, one or another ethnic group shows higher performance in comparison to the other.

The analysis of the mean performance scores of each Brazilian federal state independently indicates that in some states (but not in the majority) the gaps between ethnic groups are statistically significant in both very low and very high levels of parental education (See Tables S4 and S5 for all statistical results). In the case of mathematics, in the two states (MG and SP) that statistically significant differences are observed for all combinations of ethnic groups, one (MG) shows that the gap slightly decreased for higher education level (in comparison to the lower education level) whereas in the other (SP) the gap increased for both White-Black and White-Pardo comparisons. The comparison between White and Asian students indicate that the gap slightly increased for higher parental education in favor of Whites. However, for higher parental education levels, the gaps are higher and favor Asian students in some states (PR and SP) and White students in some others (MG and RJ). The differences (or gaps) are not statistically significant for all cases of Indigenous students but in one case of very low education, in which the gap is relatively large favoring White students. In the case of writing, either the gap decreased for White-Black and White-Pardo from very low to very high parental education level in MG state or remained approximately the same in the case of SP state.

## 4. Discussion

The achievement gap measures the difference of performance between groups of students that share some characteristics. Achievement gaps between ethnic groups as estimated through formal assessments exist in several countries but the gap size is not consistent across countries and groups [11,12,13,14]. In this paper, we study the Brazilian context by analyzing data of a national exam performed in 2014. Our analysis shows variation of achievement gaps around the country without a clear dominant group although White students tend to perform better in some contexts. For students living in low-income households, there are generally no statistically significant gaps and sometimes only small (typically bellow 6%, but in particular cases varying from 1.7% to 23.1%) statistically significant gaps are observed between the various ethnic groups. The statistical analysis indicates increasing achievement gaps between ethnic groups at higher household income levels for Sao Paulo students but these gaps are not statistically significant for the other regions of the country. In Sao Paulo, Asian students score best in mathematics, followed by White, Pardo, Black and then Indigenous students. In writing, Whites score best, followed by Asians, Blacks, Pardos and Indigenous. Household income positively affect the scores of all ethnic groups, particularly in mathematics but also in writing. The formal educational level of parents also have positive impact on the average scores of students of all ethnic groups. Contrastingly, it does not increase the gap between Whites and Blacks, and between Whites and Pardos in writing in Sao Paulo but does decrease these gaps in the MG state (one of the few states where gaps are statistically significant). For mathematics, the gaps between the groups increase in Sao Paulo but also decrease slightly in MG (with larger decrease between Whites and Blacks). In the other federal states, no statistically significant gaps are observed.

Altogether, these results provide a complex picture that makes difficult to identify particular factors increasing or decreasing gaps in the different geographical regions of Brazil, especially because in most states gaps are not statistically significant, i.e., the performance of ethnic students is similar. It is important to emphasize the cross-sectional nature of our data, meaning that we cannot make statistical causal links of factors driving the increase in achievement gaps based solely on this data set but we can identify correlations between independent and dependent variables. Our results suggest that socio-economic and environmental factors, such as poverty, lack of access to extra-curricular activities, malnutrition and lack of basic infrastructure, related to health and safety [42,43], are relevant variables that negatively affect the performance of students [44,45,46] irrespective of their ethnicity in the Brazilian context. These factors potentially have more weight than cognitive abilities driven by biological differences. If performance, as group, was mostly dependent on genetics or other biological traits, one would observe consistently similar gaps between ethnic groups irrespective of household income or parental education for all cases. The results of course do not reject that genetics, together with the environment, may affect individual intellectual abilities [47,48,49]. Given our results, we may conclude however that “speciation” was not sufficiently strong to distinguish intellectual abilities of ethnic groups.

The socio-economic, geographic and political context of Brazil is different from the USA and other Latin American countries, although they share some historical similarities in terms of immigration. Brazil is a distinct society with a relatively modern developing economy and historically witnessed massive immigration from various countries, internal migration from poorer to richer regions, low social fluidity, and high social and economic inequalities, with strong power relations between rich and poor involving a white economic elite that have persisted for several decades despite recent progresses. We argue that the observed gaps between ethnic groups are likely a result of historical developments in the country that affected ethnic groups differently due to these barriers between socio-economic classes. The current household income, for example, is a timely measure and does not reflect these historical influences that steady, or absence of steady (i.e., traditional vs. new “rich” families), finances might have in both the family social context (e.g., friends, neighborhoods) and the overall access of education and culture (e.g., extra-curricular activities, travel, entertainment). In Brazil, official educational policies began to look at different backgrounds and intellectual abilities after the new federal constitution of 1988 while constructivism pedagogy started to be implement at schools across the country [50,51]. Before this time, public education focused on preparing the working class to develop technical skills. Vocational schools for example were popular in urban areas [50] but also a large fraction of the population was still living in, or leaving, the rural areas that had limited or no access to formal education. The development of higher cognitive abilities and skills such as creativity, arts, interpretation, or critical analysis, were mostly available to those on upper classes, living in urban areas, through extra-curricular activities in private schools [51,52]. The parents of the students performing the examination analyzed in this study were affected by this historical period since their school age was during the 1980s and 1990s. As a consequence, a whole generation of lower income people, dependent on public services, was raised without proper access to quality education. This generation that became parents could not provide, later on, a proper intellectual environment to support their children to excel at school or could not socially influence them as well-educated role models. Full migration away from the technical-oriented education (that focuses on lower cognitive functions according to Bloom’s taxonomy, as for example the reproduction of protocols [53]) still faces structural challenges in the country such as lack of formal training of teachers, poor availability of education resources, e.g., computers, Internet or other materials, and teacher’s resistance to change in favor of new pedagogic inclusive theories [54]. Students without family support and opportunities to enroll on private or non-governmental extra curricular activities have remained in disadvantage and could not develop higher cognitive skills; as a consequence, these students are expected to perform worse on average than their higher income peers.

Brazilian students of Asian ancestry belonging to higher income groups achieve the best scores for mathematics. Similarly, there is a relatively stronger impact on performance of increasing parental education of Asian students. This may be related to the cultural background and attitudes of Asian families. The Asian migration to Brazil started in the early 20th century with the Japanese and then shifted towards the Koreans and Chinese in the 1980s and 1990s. All these groups have a pronounced heritage identity with strong spatial clustering, sense of community, relatively few inter-ethnic marriages and thus few mixed-descendants [55] which likely contribute to sustain the high expectations of student performance [56]. Mathematics, more than writing, requires hard-work training and is popularly associated with higher levels of intelligence and professional success, encouraging higher expectations in groups that value such characteristics. This cultural homogeneity (or social clustering and consequently social pressure) is not as strongly observed for Blacks and Whites in Brazil as observed for example in the USA. The large percentage of “mixed” populations in Brazil, without clear cultural borders, (i.e., Pardos) linking White, Black or Pardo parents result in a highly heterogeneous society where ethnic identity dilutes and thus peer-pressure becomes weaker although the sense of ethnic group exists among whites and blacks [57]. At the same time, Brazil has an European-centric educational system, where much attention is given to history and culture of Europe, to which White students directly relate, feeling empowered and motivated, sometimes developing a sense of superiority over other ethnic groups particularly Black and Indigenous populations. In comparison to northern states, e.g., Amazonas, where Pardos prevail, the European white identity is particularly stronger in southern states, e.g., Sao Paulo, where European immigration ceased later. On the other hand, Blacks and Pardos are historically stigmatized and linked to physical work, criminality and poverty, not to intellectual champions. Efforts aiming to decrease this content bias include recent federal laws (11.645/08 from 2008) obliging teaching of African history and culture, Afro-Brazilian culture and Indigenous history in Brazilian schools. The implementation of such regulations is however relatively slow and faces some resistance from part of the population, still influenced by colonial times when African and Indigenous populations were marginalized and considered of less importance [58,59].

Within this context, we reflect on potential reasons affecting the increase in the gap between White and Black students. Black families that are rich now were poorer in previous generations [60]. Consequently, although these black parents can now provide extra educational resources to their children, they remain immersed, to some extent, in a less favored intellectual environment through social ties with relatives or friends that are in disadvantage positions or still have less access to quality education. Rich white families may have also experienced this socio-economic trajectory but likely to a lesser extent given that black people have been disproportionately poorer over generations [60]. There is thus a positive feedback here. We argue that the next generation of Black students, coming from rich and educated families, will have a more similar performance to their White peers. In the United States for example, the gap has been decreasing over time [61]. It is however difficult to separate this mechanism of climbing social classes and state inclusive interventions. Data on these families social context (e.g., their preferential social interactions) and their past income (i.e., income of the parents of the current parents) would allow us to test the effect of social class mobility. The same reasoning also applies to Indigenous students whose previous generations were mostly living in rural areas and now live in urban centers [60]. In the case of poor students, we do not observe such phenomenon because families that are currently poor were poor in previous generations. Naturally, some mobility between social classes occurs but relatively few parents that were rich in the past became very poor later.

The results also show that Pardo students perform in-between Blacks and Whites, but closer to Blacks. In fact, Pardos have typically both white and black ancestry (mixed over several generations) but generally speaking have not been as poor as Blacks and have relatively less economic restrictions [60]. Previous research in the USA context showed, for example, that Black and Mixed race children adopted and raised by White families score better than the average of Black students raised by Black parents [62]. It was also observed that scores from these adopted children tended to fall during adolescence because their socio-cultural environment moved towards a Black-oriented context (i.e., other black friends, music and role models), supporting that cultural identification and peer-encouragement play a key role on learning. It is important to highlight as well that stigma and power relations of White against Black (or Indigenous) people contributes to create less expectation among Black (and Indigenous) students, suppressing their motivation by portraying them as second class citizens [63]. Therefore, even at similar social classes, expectations are generally lower for Black and Indigenous students. As mentioned above, the same effect explains the higher performance of Asians, whose parents and social environment push them for higher performance.

Our study focuses on public education where the range of economic and cultural backgrounds is larger than in private schools. This is expected to affect performance of all ethnic groups and enlarge gaps since within the economic elite, cultural identity segregates students. In private schools, the smaller variance of students imply that Blacks and Pardos are more mixed with White students; since they are minority, this mixing likely affect their scores positively and decreases gaps. One limitation of our study is that sample sizes in private schools are too small, if not zero, making it impossible to test this hypothesis with our data. Furthermore, the relatively small sample size in some categories also causes larger variations of performance scores that potentially affect the statistical significance of gaps. Although Welch’s *t*-test is robust for small sample sizes and large variances, this could have affected the results. A major limitation however is that we analyzed cross-sectional data. It would be interesting to follow up if such gaps have persisted over the years, particularly to consider different parental generations and the persistent effects of education and income in various generations. Finally, we were unable to breakdown the location of the schools and student’s residence address. Such fine-resolution analysis could provide a better understanding of the social context on students’ performance.

## 5. Conclusions

Achievement gaps between students of different ethnic backgrounds have been observed in various countries with multi-ethnic populations. Brazil is a representative middle-income country that struggles to provide free quality education to its geographically spread and socio-economic diverse population. Our analysis provides evidence that socio-economic variables play a significant role in student’s performance irrespective of ethnic background and genetic factors may have little or no effect on group performance. We also argue that recent historical developments may have promoted the relative advantage of White students in some contexts. Our results encourage the design of affirmative actions and tailored policies targeting the improvement of well-being, health, work opportunities, income and family education to students in low-income settings irrespective of their ethnic background. We further recommend target policies towards Black, Pardo and Indigenous students belonging to higher income families to provide them socio-cultural conditions to compensate potential historical disadvantages. Finally, increasing social integration of ethnic groups and exposing those disadvantaged students to successful role models may be a way forward to increase homogenization of performance scores to higher levels by social influence. Research targeting students in higher-income families could bring further insights on the driving forces behind their relatively lower (or higher) performance on such intellectual assessments.

## Figures and Tables

**Table 1:** Difference in the mean performance scores (Δ) between ethnic categories and statistical significance of this difference (*p*-value) for six household income levels (G1 to G6). The ethnic categories are White (W), Black (B), Pardo (P), Asian (A) and Indigenous (I). All means that all ethnic categories are compared together. Dark gray cells highlight cases where p<0.01 and “-” indicates that no statistics were taken because of the sample size.

	**G1**			**G2**			**G3**			**G4**			G5			**G6**		
	**Δ(%)**	**F**	**p-Value**	**Δ(%)**	**F**	**p-Value**	**Δ(%)**	**F**	**p-Value**	**Δ(%)**	**F**	**p-Value**	Δ(%)	**F**	**p-Value**	**Δ(%)**	**F**	**p-Value**
**Sao Paulo—Mathematics**																		
All		$F_{4,389}=11.52$	<0.01		$F_{4,1271}=43.93$	<0.01		$F_{4,792}=78.45$	<0.01		$F_{4,183}=28.44$	<0.01		$F_{3,278}=16.31$	<0.01		$F_{3,226}=17.16$	<0.01
W-B	2.0	$F_{4,2848}=4.24$	<0.01	2.2	$F_{4,6728}=7.70$	<0.01	4.0	$F_{4,4180}=11$	<0.01	3.6	$F_{4,1095}=5.55$	<0.01	7.3	$F_{4,296}=5.62$	<0.01	6.5	$F_{4,166}=3.50$	<0.01
W-P	2.1	$F_{4,11763}=6.43$	<0.01	2.3	$F_{4,35744}=12.21$	<0.01	3.0	$F_{4,25254}=14.14$	<0.01	3.6	$F_{4,6585}=9.08$	<0.01	3.5	$F_{4,1353}=4.07$	<0.01	3.9	$F_{4,887}=3.86$	<0.01
W-A	1.0	$F_{4,159}=0.79$	0.43	−0.9	$F_{4,559}=-0.99$	0.32	−4.8	$F_{4,538}=-5.15$	<0.01	−6.0	$F_{4,178}=-3.38$	<0.01	−5.9	$F_{4,74}=-2.20$	0.03	−14.5	$F_{4,72}=-2.88$	<0.01
W-I	1.8	$F_{4,75}=0.96$	0.34	2.9	$F_{4,238}=2.56$	0.01	1.6	$F_{4,127}=0.78$	0.44	3.0	$F_{4,27}=0.92$	0.36	-	-	-	-	-	-
**Sao Paulo—Writing**																		
All		$F_{4,388}=9.37$	<0.01		$F_{4,1271}=31.49$	<0.01		$F_{4,794}=39.79$	<0.01		$F_{4,183}=11.00$	<0.01		$F_{3,277}=6.01$	<0.01		$F_{3,230}=9.64$	<0.01
W-B	3.4	$F_{4,2592}=3.73$	<0.01	3.5	$F_{4,6259}=6.87$	<0.01	3.6	$F_{4,4012}=6.63$	<0.01	3.8	$F_{4,1037}=3.79$	<0.01	1.3	$F_{4,276}=0.68$	0.50	9.0	$F_{4,165}=3.98$	<0.01
W-P	3.4	$F_{4,11457}=5.75$	<0.01	3.1	$F_{4,33893}=10.00$	<0.01	3.8	$F_{4,23202}=11.37$	<0.01	2.8	$F_{4,5952}=5.07$	<0.01	4.7	$F_{4,1361}=4.23$	<0.01	2.6	$F_{4,814}=-2.07$	0.04
W-A	2.2	$F_{4,157}=0.81$	0.42	2.3	$F_{4,565}=1.85$	0.06	−2.0	$F_{4,543}=-1.66$	0.10	−4.8	$F_{4,181}=-2.46$	0.01	−0.3	$F_{4,75}=-0.11$	0.91	−7.3	$F_{4,72}=-2.88$	<0.01
W-I	1.3	$F_{4,75}=0.35$	0.72	6.2	$F_{4,236}=2.94$	<0.01	5.4	$F_{4,127}=2.12$	0.04	4.6	$F_{4,27}=1.07$	0.30	-	-	-	-	-	-
**Amazonas—Mathematics**																		
All		$F_{4,109}=0.59$	0.67		$F_{4,139}=2.57$	0.04		$F_{4,56}=0.34$	0.85		$F_{4,39}=0.91$	0.41		-	-		-	-
W-B	−1.0	$F_{4,203}=-0.57$	0.57	−3.8	$F_{4,166}=-1.80$	0.07	−0.4	$F_{4,96}=-0.16$	0.88	−1.7	$F_{4,26}=-0.37$	0.72	-	-	-	-	-	-
W-P	0.3	$F_{4,459}=0.27$	0.79	1.3	$F_{4,655}=1.29$	0.20	0.1	$F_{4,396}=0.05$	0.96	2.8	$F_{4,138}=0.99$	0.32	2.7	$F_{4,83}=0.54$	0.59	0.6	$F_{4,107}=0.14$	0.89
W-A	−2.2	$F_{4,49.80}=-0.89$	0.38	−0.2	$F_{4,71}=-0.06$	0.95	−3.5	$F_{4,31}=-0.81$	0.43	-	-	-	-	-	-	-	-	-
W-I	−2.0	$F_{4,34}=0.68$	0.50	5.3	$F_{4,39}=1.80$	0.08	4.1	$F_{4,13}=0.77$	0.45	-	-	-	-	-	-	-	-	-
**Amazonas—Writing**																		
All		$F_{4,}=$	<0.01		$F_{4,}=$	0.07		$F_{4,}=$	0.03		$F_{2,}=$	0.54		-	-		-	-
W-B	5.0	$F_{1,183}=1.23$	0.22	3.2	$F_{1,172}=0.92$	0.36	−5.5	$F_{1,90}=-1.21$	0.23	7.3	$F_{1,20}=1.04$	0.31	-	-	-	-	-	-
W-P	6.2	$F_{1,477}=2.79$	<0.01	2.4	$F_{1,707}=1.42$	0.16	−2.8	$F_{1,380}=-1.10$	0.27	0	$F_{1,154}=-0.01$	0.99	6.3	$F_{1,87}=0.93$	0.36	1.0	$F_{1,98}=0.22$	0.83
W-A	−7.3	$F_{1,49}=-1.45$	0.15	4.8	$F_{1,64}=-0.97$	0.33	−1.2	$F_{1,380}=-0.16$	0.87	-	-	-	-	-	-	-	-	-
W-I	4.0	$F_{1,33}=0.62$	0.54	18.4	$F_{1,37}=2.77$	<0.01	−17.9	$F_{1,31}=-3.40$	<0.01	-	-	-	-	-	-	-	-	-

**Table 2:** Difference in the mean performance scores (Δ) between ethnic categories and statistical significance of this difference (*p*-value) for six parental education levels (G1 to G6). The ethnic categories are White (W), Black (B), Pardo (P), Asian (A) and Indigenous (I). All means that all ethnic categories are compared together. Dark gray cells highlight cases where p<0.01 and “-” indicates that no statistics were taken because of the sample size.

	**G1**			**G2**			**G3**			**G4**			G5			**G6**		
	**Δ(%)**	**F**	**p-Value**	**Δ(%)**	**F**	**p-Value**	**Δ(%)**	**F**	**p-Value**	**Δ(%)**	**F**	**p-Value**	Δ(%)	**F**	**p-Value**	**Δ(%)**	**F**	**p-Value**
**Sao Paulo—Mathematics**																		
All		$F_{2,84}=2.75$	0.07		$F_{4,246}=10.89$	<0.01		$F_{4,615}=39.68$	<0.01		$F_{4,1170}=87.07$	<0.01		$F_{4,358}=86.17$	<0.01		$F_{4,73}=19.41$	<0.01
W-B	7.4	$F_{1,63}=2.06$	0.04	2.8	$F_{1,1183}=3.98$	<0.01	3.5	$F_{1,3409}=8.71$	<0.01	3.6	$F_{1,6485}=12.48$	<0.01	6.1	$F_{1,1731}=10.86$	<0.01	6.0	$F_{1,422}=5.17$	<0.01
W-P	5.2	$F_{1,169}=1.98$	0.05	2.5	$F_{1,7068}=5.99$	<0.01	2.7	$F_{1,21940}=11.05$	<0.01	2.8	$F_{1,35611}=15.19$	<0.01	5.4	$F_{1,8120}=14.79$	<0.01	5.2	$F_{1,1673}=6.79$	<0.01
W-A	-	-	-	2.3	$F_{1,103}=1.31$	0.19	0.9	$F_{1,315}=0.80$	0.42	−4.0	$F_{1,617}=-4.59$	<0.01	−9.1	$F_{1,322}=-6.50$	<0.01	−10.7	$F_{1,62}=-3.26$	<0.01
W-I	-	-	-	4.5	$F_{1,48}=2.09$	0.04	4.3	$F_{1,108}=2.37$	0.02	1.7	$F_{1,202}=1.26$	0.21	6.0	$F_{1,54}=1.89$	0.06	11.9	$F_{1,11}=2.11$	0.06
**Sao Paulo—Writing**																		
All		$F_{2,76}=0.51$	0.60		$F_{4,244}=9.24$	<0.01		$F_{4,615}=25.55$	<0.01		$F_{4,1170}=42.33$	<0.01		$F_{4,359}=33.60$	<0.01		$F_{4,6.19}=72.99$	<0.01
W-B	1.8	$F_{1,48}=0.24$	0.81	5.7	$F_{1,1158}=4.67$	<0.01	4.7	$F_{1,3132}=6.50$	<0.01	3.4	$F_{1,6090}=7.53$	<0.01	5.7	$F_{1,1618}=6.86$	<0.01	4.4	$F_{1,404}=2.81$	<0.01
W-P	4.4	$F_{1,167}=1.00$	0.32	3.0	$F_{1,6832}=3.99$	<0.01	3.6	$F_{1,20981}=8.83$	<0.01	3.3	$F_{1,33132}=11.70$	<0.01	5.0	$F_{1,7018}=9.324.54$	<0.01	4.8	$F_{1,1485}=4.54$	<0.01
W-A	-	-	-	8.2	$F_{1,101}=2.24$	0.03	−0.9	$F_{1,317}=-0.58$	0.56	−0.4	$F_{1,622}=-0.36$	0.72	−5.2	$F_{1,327}=-3.23$	<0.01	1.5	$F_{1,62}=0.41$	0.68
W-I	-	-	-	12.9	$F_{1,47}=2.42$	0.02	5.0	$F_{1,108}=1.66$	0.10	5.3	$F_{1,202}=2.65$	<0.01	5.9	$F_{1,55}=1.66$	0.10	3.2	$F_{1,11}=0.53$	0.60
**Amazonas—Mathematics**																		
All		-	-		$F_{2,31}=2.26$	0.12		$F_{2,51}=0.28$	0.89		$F_{4,138}=4.02$	<0.01		$F_{4,51}=1.22$	0.31		$F_{2,60}=1.87$	0.16
W-B	-	-	-	−2.0	$F_{1,36}=-0.42$	0.67	1.3	$F_{1,69}=0.42$	0.68	−0.7	$F_{1,228}=-0.41$	0.69	−4.4	$F_{1,52}=-1.11$	0.27	3.3	$F_{1,33}=0.76$	0.45
W-P	-	-	-	4.6	$F_{1,43}=1.37$	0.18	−1.0	$F_{1,210}=-0.61$	0.55	2.7	$F_{1,774}=2.87$	<0.01	2.3	$F_{1,316}=1.27$	0.2	4.4	$F_{1,231}=1.94$	0.05
W-A	-	-	-	-	-	-	−2.3	$F_{1,21}=-0.53$	0.60	−0.6	$F_{1,91}=-0.30$	0.77	−2.4	$F_{1,18}=-0.42$	0.68	-	-	-
W-I	-	-	-	-	-	-	−0.1	$F_{1,21}=-0.03$	0.98	6.4	$F_{1,34}=2.19$	0.03	3.4	$F_{1,18}=0.56$	0.58	-	-	-
**Amazonas—Writing**																		
All		-	-		$F_{2,30.7}=0.08$	0.93		$F_{2,51.6}=0.55$	0.70		$F_{4,138}=3.51$	<0.01		$F_{4,51}=0.60$	0.66		$F_{2,60}=2.41$	0.10
W-B	-	-	-	4.6	$F_{1,34}=0.37$	0.72	5.5	$F_{1,62}=0.83$	0.41	0.3	$F_{1,213}=0.12$	0.90	9.4	$F_{1,45}=1.29$	0.20	−4.0	$F_{1,31}=-0.72$	0.47
W-P	-	-	-	2.8	$F_{1,42}=0.34$	0.73	3.2	$F_{1,225}=0.96$	0.34	4.0	$F_{1,857}=2.52$	0.01	−0.7	$F_{1,364}=-0.29$	0.77	4.8	$F_{1,253}=1.72$	0.09
W-A	-	-	-	-	-	-	−13.7	$F_{1,18}=1.06$	0.30	−5.1	$F_{1,90}=-1.54$	0.13	3.3	$F_{1,17}=0.41$	0.69	-	-	-
W-I	-	-	-	-	-	-	−1.1	$F_{1,22}=-0.17$	0.87	5.4	$F_{1,31}=0.85$	0.40	3.8	$F_{1,17}=0.39$	0.70	-	-	-

## Supplementary Materials

The following are available online at https://www.mdpi.com/2079-3200/7/1/7/s1, Table S1: Keywords for the inclusion criteria, values and brief description, Table S2: The mean performance scores and *p*-values for household income levels in mathematics, Table S3: The mean performance scores and *p*-values for household income levels in writing, Table S4: The mean performance scores and *p*-values for parental education levels in mathematics, Table S5: The mean performance scores and *p*-values for parental education levels in writing.
