# Supplementary material for: Assessing Student’s Achievement Gaps between Ethnic Groups in Brazil
Source: J Intell. 2019 Feb 20;7(1):7. doi: 10.3390/jintelligence7010007 (PMC6526449; doi:10.3390/jintelligence7010007)
Supplement: Supplementary file 1 [file jintelligence-07-00007-s001.zip › jintelligence-397368-SI.pdf]

J. Intell. 2019, 7, 7; doi:10.3390/jintelligence7010007

Supplementary Information: Assessing student's achievement gap between ethnic groups in Brazil

Luis E C Rocha*
Centre for Business Network Analysis, University of Greenwich, London, UK

Luana F Nascimento†
Radiation Protection Dosimetry and Calibration group, Belgian Nuclear Research Center, Mol, Belgium

(Dated: February 20, 2019)

* luis.rocha@gre.ac.uk
† ldfnasci@sckcen.be

TABLE S1. **Keywords for the inclusion criteria, values and brief description. Keywords as they appear in the original data set.**

CODE	VALUE	BRIEF DESCRIPTION
ID_DEPENDENCIA_ADM_ESC	1 AND 2 AND 3	school is public management
ID_LOCALIZACAO_ESC	1	school is urban
SIT_FUNC_ESC	1	school is active
IDADE	17	student age
NACIONALIDADE	1	student is born in Brazil
ST_CONCLUSAO	2	student will graduate in 2013
TP_ESCOLA	1	school is public
IN_TP_ENSINO	1	school is not for special students
TP_ESTADO_CIVIL	0	student is single
IN_BAIXA_VISAO	0	student has no low vision
IN_CEGUEIRA	0	student is not blind
IN_SURDEZ	0	student is not deaf
IN_DEFICIENCIA_AUDITIVA	0	student has no auditory disability
IN_SURDO_CEGUEIRA	0	student is not deaf and not blind
IN_DEFICIENCIA_FISICA	0	student has no physical disability
IN_DEFICIENCIA_MENTAL	0	student has no mental disability
IN_DEFICIT_ATENCAO	0	student has no attention deficit
IN_DISLEXIA	0	student has not dyslexia
IN_GESTANTE	0	student is not pregnant
IN_LACTANTE	0	student is not lactating
IN_IDOSO	0	elderly student, 60 or above
IN_AUTISMO	0	student is not autistic
IN_BRAILLE	0	student does not need exam in Braille
IN_AMPLIADA_24	0	student does not need exam with larger fonts
IN_AMPLIADA_18	0	student does not need exam with larger fonts
IN_LEDOR	0	student does not need an auxiliary person to read and write the exam
IN_ACESSO	0	student does not need a class with special access
IN_TRANSCRICAO	0	student does not need an auxiliary person to transcribe the text
IN_LIBRAS	0	student does not need an auxiliary person for sign language
IN_LEITURA_LABIAL	0	student does not need an auxiliary person for lip reading
IN_MESA_CADEIRA_RODAS	0	student does not need a desk for wheelchair
IN_MESA_CADEIRA_SEPARADA	0	student does not need separated desk and chair
IN_APOIO_PERNA	0	student does not need an auxiliary device to rest his/her legs
IN_GUIA_INTERPRETE	0	student does not need an auxiliary person for translation
IN_PRESENCA_CN	1	student participated in the natural sciences examination
IN_PRESENCA_CH	1	student participated in the human sciences examination
IN_PRESENCA_LC	1	student participated in the languages examination
IN_PRESENCA_MT	1	student participated in the mathematics examination
Q004	1 AND 2 AND 3<br>4 AND 5 AND 6	student lives in a household with 6 or less people
Q006	B	student lives in an urban area

TABLE S2. **The gap (Δ) in the performance scores between ethnic groups and respective statistical significance (*p*-values) for household income levels in mathematics. Very low income corresponds to G1 in the main text, and very high income corresponds to G6. Cases of $p < .01$ are highlighted. Ethnic groups correspond to W: White; B: Black; P: Pardo; A: Asian; I: Indigenous; All: All ethnic groups compared simultaneously.**

		Very low income					Very high income				
Brazilian State		All	W-B	W-P	W-A	W-I	All	W-B	W-P	W-A	W-I
AC	Δ(%)		0.9	-2.0	-3.3	-		-	-	-	-
	p-value	.40	.73	.29	.46	-	-	-	-	-	-
AL	Δ(%)		-0.2	1.3	-1.9	4.5		-	-	-	-
	p-value	.47	.91	.29	.43	.43	-	-	-	-	-
AP	Δ(%)		0.1	-0.3	-7.0	-		-	-	-	-
	p-value	.43	.96	.86	.13	-	-	-	-	-	-
AM	Δ(%)		-1.0	0.3	-2.2	-2.0		-	0.6	-	-
	p-value	.67	.57	.79	.38	.50	-	-	.89	-	-
BA	Δ(%)		1.4	1.7	1.2	2.6		-	2.6	-	-
	p-value	.09	.04	< .01	.35	.17	-	-	.71	-	-
CE	Δ(%)		0.6	2.7	-0.9	1.2		-	12.0	-	-
	p-value	< .01	.40	< .01	.53	.64	-	-	.25	-	-
DF	Δ(%)		3.7	3.3	-0.1	11.0		10.3	5.4	-	-
	p-value	.11	.07	.03	.98	-	< .01	.01	.01	-	-
ES	Δ(%)		4.1	1.2	0.1	-4.2		-	2.0	-	-
	p-value	.08	< .01	.23	.97	.34	-	-	.65	-	-
GO	Δ(%)		1.0	1.5	0.9	2.3		-2.3	-0.2	-	-
	p-value	.35	.33	.04	.60	.48	.94	.73	.94	-	-
MA	Δ(%)		0.4	1.3	-1.3	6.9		-	-	-	-
	p-value	.05	.59	.05	.48	.04	-	-	-	-	-
MT	Δ(%)		1.1	1.8	4.8	-		-	0.2	-	-
	p-value	.31	.45	.09	.25	-	-	-	.82	-	-
MS	Δ(%)		-0.6	1.9	-1.4	-		-	-1.1	-	-
	p-value	.21	.77	.06	.72	-	-	-	.84	-	-
MG	Δ(%)		2.9	2.6	3.4	3.3		5.2	2.0	13.7	-
	p-value	< .01	< .01	< .01	< .01	.06	.05	.05	.21	.05	-
PR	Δ(%)		4.1	1.1	1.3	-		1.0	0	-11.0	-
	p-value	.01	< .01	.09	.60	-	.46	.89	.99	.13	-
PB	Δ(%)		2.9	1.6	1.9	3.6		-	-	-	-
	p-value	.10	.01	.03	.30	.29	-	-	-	-	-
PA	Δ(%)		-1.7	0.1	2.0	2.4		-	2.9	-	-
	p-value	.28	.13	.93	.37	.58	-	-	.58	-	-
PE	Δ(%)		1.2	1.2	1.8	3.7		-	4.6	-	-
	p-value	.07	.09	.01	.16	.09	-	-	.36	-	-
PI	Δ(%)		1.1	1.6	-0.6	2.8		-	-	-	-
	p-value	.48	.42	.10	.79	.63	-	-	-	-	-
RJ	Δ(%)		4.5	3.2	1.4	5.2		11.2	7.0	-	-
	p-value	< .01	< .01	< .01	.51	.24	< .01	.01	< .01	-	-
RN	Δ(%)		0	0.9	2.5	0		-	-	-	-
	p-value	.71	.99	.28	.28	.99	-	-	-	-	-
RO	Δ(%)		-4.5	-2.0	-1.6	-		-	5.3	-	-
	p-value	.23	.04	.19	.60	-	-	-	.31	-	-
RR	Δ(%)		0.4	0.5	-	-		-	-	-	-
	p-value	.98	.93	.84	-	-	-	-	-	-	-
RS	Δ(%)		3.6	2.1	0.6	-		6.2	2.4	-	-
	p-value	< .01	< .01	.01	.85	-	.44	.26	.51	-	-
SC	Δ(%)		1.4	2.7	-3.4	-		-	3.4	-	-
	p-value	.10	.47	.02	.39	-	-	-	.38	-	-
SE	Δ(%)		-1.0	-0.8	0.4	3.0		-	-1-	-	-
	p-value	.83	.58	.58	.89	.45	-	-	-	-	-
SP	Δ(%)		2.0	2.1	1.0	1.8		6.5	3.9	-14.5	-
	p-value	< .01	< .01	< .01	.43	.34	< .01	< .01	< .01	< .01	-
TO	Δ(%)		2.6	0.3	-3.4	-		-7.2	-7.4	-	-
	p-value	.10	.10	.81	.21	-	.44	.33	.21	-	-

TABLE S3. **The gap (Δ) in the performance scores between ethnic groups and respective statistical significance (p-values) for household income levels in writing. Very low income corresponds to G1 in the main text, and very high income corresponds to G6. Cases of $p < .01$ are highlighted. Ethnic groups correspond to W: White; B: Black; P: Pardo; A: Asian; I: Indigenous; All: All ethnic groups compared simultaneously.**

		Very low income					Very high income				
Brazilian State		All	W-B	W-P	W-A	W-I	All	W-B	W-P	W-A	W-I
AC	Δ(%)		-4.4	-0.2	-11.1	-		-	-	-	-
	p-value	.10	.39	.96	.04	-	-	-	-	-	-
AL	Δ(%)		2.6	1.9	-0.6	2.5		-	-	-	-
	p-value	.93	.52	.49	.91	.84	-	-	-	-	-
AP	Δ(%)		13.5	8.8	-8.6	-		-	-	-	-
	p-value	< .01	< .01	.02	.28	-	-	-	-	-	-
AM	Δ(%)		5.0	6.2	-7.3	4.0		-	1.0	-	-
	p-value	< .01	.22	< .01	.15	.54	-	-	.83	-	-
BA	Δ(%)		3.6	3.0	-0.6	16.8		-	-2.6	-	-
	p-value	< .01	.01	.02	.83	< .01	-	-	.70	-	-
CE	Δ(%)		1.6	5.6	0.6	4.7		-	7.3	-	-
	p-value	< .01	.27	< .01	.81	.36	-	-	.56	-	-
DF	Δ(%)		0.1	4.1	1.9	-		10.0	5.6	-	-
	p-value	.35	.98	.11	.73	-	.01	.01	.02	-	-
ES	Δ(%)		7.5	3.9	8.9	4.3		-	4.2	-	-
	p-value	.09	.01	.03	.24	.58	-	-	.46	-	-
GO	Δ(%)		1.6	3.2	-2.4	1.1		-1.7	4.2	-	-
	p-value	.13	.47	.04	.41	.88	.59	.83	.34	-	-
MA	Δ(%)		1.3	4.2	-2.8	-5.0		-	-	-	-
	p-value	.03	.52	< .01	.51	.60	-	-	-	-	-
MT	Δ(%)		9.0	3.7	23.1	-		-	-	-	-
	p-value	< .01	< .01	.09	< .01	-	-	-	-	-	-
MS	Δ(%)		1.3	3.0	23.7	-		-	0.2	-	-
	p-value	.11	.72	.12	.05	-	-	-	.98	-	-
MG	Δ(%)		4.7	4.9	4.6	6.0		2.9	2.4	11.2	-
	p-value	< .01	< .01	< .01	.04	.09	.20	.45	.23	.07	-
PR	Δ(%)		9.3	2.9	-6.1	-		7.8	7.3	-9.8	-
	p-value	< .01	< .01	.03	.20	-	.11	.39	.05	.19	-
PB	Δ(%)		2.4	1.6	4.3	7.8		-	-	-	-
	p-value	.56	.31	.30	.31	.25	-	-	-	-	-
PA	Δ(%)		-0.8	2.2	-1.8	4.9		-	-0.3	-	-
	p-value	.28	.70	.17	.69	.65	-	-	.98	-	-
PE	Δ(%)		6.3	5.2	8.0	5.5		-	9.2	-	-
	p-value	< .01	< .01	< .01	< .01	.17	-	-	.11	-	-
PI	Δ(%)		4.9	2.8	1.6	8.1		-	-	-	-
	p-value	.49	.08	.18	.73	.44	-	-	-	-	-
RJ	Δ(%)		5.5	6.1	-2.5	-0.7		5.2	4.0	-	-
	p-value	< .01	< .01	< .01	.44	.92	.14	.18	.09	-	-
RN	Δ(%)		4.1	3.0	1.5	-3.8		-	-	-	-
	p-value	.36	.12	.08	.77	.63	-	-	-	-	-
RO	Δ(%)		-3.3	0.3	1.2	-		-	-6.7	-	-
	p-value	.89	.51	.93	.90	-	-	-	.54	-	-
RR	Δ(%)		10.0	12.5	-	-		-	-	-	-
	p-value	.11	.22	.04	-	-	-	-	-	-	-
RS	Δ(%)		2.4	2.7	4.8	-		15.9	-3.6	-	-
	p-value	.23	.24	.08	.44	-	.12	.08	.31	-	-
SC	Δ(%)		-1.2	0	-0.2	-		-	-3.4	-	-
	p-value	.99	.71	.99	.94	-	.99	-	.48	-	-
SE	Δ(%)		-3.3	-5.7	-9.1	9.0		-	-	-	-
	p-value	.20	.39	.06	.15	.43	-	-	-	-	-
SP	Δ(%)		3.4	3.4	2.2	1.3		9.0	2.6	-7.4	-
	p-value	< .01	< .01	< .01	.42	.72	< .01	< .01	.04	< .01	-
TO	Δ(%)		5.4	1.3	0.9	-		8.4	6.7	-	-
	p-value	.58	.21	.69	.89	-	.40	.19	.28	-	-

TABLE S4. **The gap (Δ) in the performance scores between ethnic groups and respective statistical significance (*p*-values) for parental education levels in mathematics. Very low education corresponds to G2 in the main text, and very high education corresponds to G5. Cases of $p < .01$ are highlighted. Ethnic groups correspond to W: White; B: Black; P: Pardo; A: Asian; I: Indigenous; All: All ethnic groups compared simultaneously.**

		Very low education					Very high education				
Brazilian State		All	W-B	W-P	W-A	W-I	All	W-B	W-P	W-A	W-I
AC	Δ(%)		0.6	5.4	-	-		2.6	-3.0	-	-
	p-value	.2	.90	.15	-	-	.11	.44	.17	-	-
AL	Δ(%)		8.2	4.0	2.5	-		-3.4	3.7	-	-
	p-value	.08	< .01	.07	.61	-	.27	.56	.23	-	-
AP	Δ(%)		-	-1.1	-	-		-0.5	-2.0	-	-
	p-value	-	-	.88	-	-	.65	.88	0.39	-	-
AM	Δ(%)		-0.1	5.6	-	-		-1.4	3.2	-4.7	8.1
	p-value	.09	.98	.08	-	-	.04	.62	.02	.34	.10
BA	Δ(%)		3.7	2.4	3.2	-1.6		0.1	0	3.9	5.0
	p-value	.14	.01	.06	.25	.73	.54	.54	.98	.19	.35
CE	Δ(%)		-0.5	3.0	-2.5	14.0		-0.1	0.9	-1.2	4.9
	p-value	< .01	.74	< 0.01	.32	< .01	.75	.97	.49	.74	.27
DF	Δ(%)		-1.0	3.8	-	-		6.2	4.4	2.8	-
	p-value	.2	.80	.17	-	-	< .01	< .01	< .01	0.44	-
ES	Δ(%)		6.2	4.5	-	-		4.8	2.6	4.9	-
	p-value	< .01	< .01	< .01	-	-	.06	.02	.04	.34	-
GO	Δ(%)		4.1	1.4	1.8	-		3.5	1.8	2.8	-
	p-value	.18	.03	.29	.43	-	.07	.02	.05	.20	-
MA	Δ(%)		2.8	0.8	2.9	-		-1.7	0.9	2.6	-
	p-value	.46	.14	.57	.43	-	.48	.39	.46	.57	-
MT	Δ(%)		0	3.8	-	-		4.2	2.3	2.4	-
	p-value	.08	.99	.04	-	-	.04	< .01	.02	.68	-
MS	Δ(%)		-3.2	4.3	-	-		2.7	2.7	-3.2	-
	p-value	.04	.36	.04	-	-	.14	.36	.03	.55	-
MG	Δ(%)		6.3	3.8	4.8	4.9		4.1	3.3	5.2	9.5
	p-value	< .01	< .01	< .01	< .01	.13	< .01	< .01	< .01	< .01	.02
PR	Δ(%)		3.9	1.7	-5.6	-		5.7	3.9	-8.4	-
	p-value	.01	.01	.02	.29	-	< .01	< .01	< .01	< .01	-
PB	Δ(%)		-0.8	-0.3	-1.0	7.9		4.9	5.3	11.0	-
	p-value	.14	.74	.82	.78	.03	. < 01	0.10	< .01	.01	-
PA	Δ(%)		1.7	1.6	-	-		-1.4	0.6	1.2	-
	p-value	.75	.54	.45	-	-	.76	.53	.68	.79	-
PE	Δ(%)		2.7	0.6	0.3	-2.4		6.0	1.9	4.9	9.2
	p-value	.56	.10	.57	.90	.70	< .01	< .01	.09	.16	.02
PI	Δ(%)		3.7	4.5	6.1	-		1.8	-1.3	-3.5	-
	p-value	.11	.20	.02	.12	-	.50	.49	.51	.51	-
RJ	Δ(%)		2.0	-0.9	4.2	-		10.0	5.9	17.2	-
	p-value	.30	.29	.56	.25	-	< .01	< .01	< .01	< .01	-
RN	Δ(%)		1.2	2.8	7.9	-		2.0	2.1	0.2	-
	p-value	.18	.68	.10	.07	-	.81	.64	.33	.98	-
RS	Δ(%)		7.8	3.1	7.9	-		8.5	3.8	5.5	-
	p-value	< .01	< .01	.01	.09	-	< .01	< .01	< .01	.21	-
RO	Δ(%)		-0.4	-0.1	-	-		-0.8	1.6	4.5	-
	p-value	-	.92	.97	-	-	.48	.80	.25	.27	-
RR	Δ(%)		-	-	-	-		-1.9	1.8	-	-
	p-value	-	-	-	-	-	.64	.68	.56	-	-
SC	Δ(%)		3.3	1.8	-1.4	-		4.8	5.6	6.8	-
	p-value	.24	.20	.11	.62	-	< .01	.07	< .01	.06	-
SE	Δ(%)		-2.8	-2.2	0.6	-		1.7	0.9	-	-
	p-value	.81	.45	.47	.90	-	.93	.71	.82	-	-
SP	Δ(%)		2.9	2.6	2.8	4.8		6.2	5.5	-9.1	7.3
	p-value	< .01	< .01	< .01	.11	.03	< .01	< .01	< .01	< .01	.01
TO	Δ(%)		4.7	0.4	-	-		3.0	5.0	2.2	-
	p-value	.24	.18	.89	-	-	.02	.16	< .01	.56	-

TABLE S5. **The gap (Δ) in the performance scores between ethnic groups and respective statistical significance (*p*-values) for parental education levels in writing. Very low education corresponds to G2 in the main text, and very high education corresponds to G5. Cases of $p < .01$ are highlighted. Ethnic groups correspond to W: White; B: Black; P: Pardo; A: Asian; I: Indigenous; All: All ethnic groups compared simultaneously.**

		Very low education					Very high education				
Brazilian State		All	W-B	W-P	W-A	W-I	All	W-B	W-P	W-A	W-I
AC	Δ(%)		-1.7	4.4	-	-		1.1	-0.1	-	-
	p-value	.57	.82	.45	-	-	0.99	0.90	0.97	-	-
AL	Δ(%)		11.5	4.8	2.1	-		-3.4	3.7	-	-
	p-value	0.41	0.09	0.35	0.85	-	0.08	0.74	0.05	-	-
AP	Δ(%)		-	-11.7	-	-		3.7	0.2	-	-
	p-value	-	-	0.43	-	-	0.72	0.50	0.96	-	-
AM	Δ(%)		-0.5	1.5	-	-		-3.9	1.5	-1.2	5.4
	p-value	.96	.97	.85	-	-	.86	.43	.41	.85	.47
BA	Δ(%)		3.2	-0.7	-9.9	15.6		-3.3	-2.1	1.8	20.3
	p-value	.12	.36	.82	.09	.23	.27	.12	.25	.67	.22
CE	Δ(%)		1.4	6.0	-4.2	-5.4		-3.3	-1.3	-7.5	-1.9
	p-value	< .01	.69	< 0.01	.40	.46	.31	.25	.46	.04	.74
DF	Δ(%)		-2.1	4.2	-	-		0.7	3.3	-0.9	-
	p-value	.32	.66	.31	-	-	.17	.77	.03	.84	-
ES	Δ(%)		9.0	2.6	-	-		3.5	2.5	11.7	-
	p-value	.09	.03	.31	-	-	.29	.19	.16	.22	-
GO	Δ(%)		5.0	6.0	7.6	-		4.0	2.7	-2.2	-
	p-value	.21	.20	.04	.14	-	.11	.09	.06	.52	-
MA	Δ(%)		-3.80	5.2	-0.7	-		-3.1	-0.4	-5.4	-
	p-value	.13	.53	.22	.95	-	.60	.33	.85	.33	-
MT	Δ(%)		5.9	3.9	-	-		-1.8	2.5	5.5	-
	p-value	.45	.33	.27	-	-	.27	.51	.14	.44	-
MS	Δ(%)		-3.9	-0.9	-	-		-2.9	4.5	1.7	-
	p-value	.78	.48	.80	-	-	.06	.42	.01	.83	-
MG	Δ(%)		8.1	5.5	2.2	13.1		4.2	3.5	5.7	15.3
	p-value	< .01	< .01	< .01	.44	.02	< .01	< .01	< .01	.06	< .01
PR	Δ(%)		7.8	2.3	-18.3	-		1.2	2.5	-9.0	-
	p-value	< .01	.03	.11	< .01	-	< .01	.58	.02	< .01	-
PB	Δ(%)		1.5	4.3	3.4	47.3		8.7	1.2	-3.0	-
	p-value	.08	.72	.18	.67	.02	.24	0.05	.66	.75	-
PA	Δ(%)		-2.8	-0.3	-	-		10.8	7.1	19.0	-
	p-value	.84	.61	.95	-	-	< .01	< .01	< .01	.02	-
PE	Δ(%)		7.1	6.2	2.7	5.9		4.9	2.6	6.3	14.6
	p-value	.07	.06	< .01	.55	.62	.06	.06	.11	.19	.03
PI	Δ(%)		3.7	4.0	9.2	-		6.7	-0.2	-4.9	-
	p-value	.81	.52	.41	.43	-	.39	.19	.95	.54	-
RJ	Δ(%)		2.3	-0.5	0.5	-		5.3	5.4	12.6	-
	p-value	.83	.45	.81	.94	-	< .01	< .01	< .01	.04	-
RN	Δ(%)		8.9	6.4	-6.3	-		0.2	2.6	-3.2	-
	p-value	.20	.18	.10	.48	-	.81	.98	.42	.69	-
RS	Δ(%)		13.2	2.6	15.6	-		9.1	0.7	8.3	-
	p-value	.02	< .01	.21	.11	-	< .01	< .01	.64	.24	-
RO	Δ(%)		-10.5	-1.5	-	-		4.9	1.9	-0.7	-
	p-value	.29	.12	.74	-	-	.64	.26	.39	.91	-
RR	Δ(%)		-	-	-	-		-4.4	11.6	-	-
	p-value	-	-	-	-	-	.01	.53	.01	-	-
SC	Δ(%)		2.4	1.3	0.2	-		5.1	6.1	0.9	-
	p-value	.89	.60	.52	.97	-	< .01	.15	< .01	.84	-
SE	Δ(%)		1.4	-5.4	-8.6	-		0.6	-5.5	-	-
	p-value	.59	.88	.43	.37	-	.39	.93	.32	-	-
SP	Δ(%)		5.6	3.0	8.4	11.9		5.5	5.1	-3.8	5.6
	p-value	< .01	< .01	< .01	.02	.03	< .01	< .01	< .01	< .01	.08
TO	Δ(%)		11.8	13.8	-	-		12.1	11.6	6.4	-
	p-value	.07	.12	.02	-	-	.02	< .01	< .01	.28	-
